# Editorial: Mutant p53 in Cancer Progression and Personalized Therapeutic Treatments

**DOI:** 10.3389/fonc.2021.740578

**Published:** 2021-08-11

**Authors:** Oleg Timofeev

**Affiliations:** Institute of Molecular Oncology, Universities of Giessen and Marburg Lung Center (UGMLC), Member of the German Center for Lung Research (DZL), Philipps-University, Marburg, Germany

**Keywords:** mutant p53, targeted therapy, tumor stroma, metabolic reprogramming, cancer stem cell

The p53 tumor suppressor is a transcriptional factor that controls a network of cellular processes essential for the maintenance of genomic integrity and prevention of malignant transformation. Ironically, p53 was initially described as an oncogene, and it took quite some time to realize that the protein found in tumors was a mutated version. Mutations in the TP53 gene are the most frequent genetic alterations detected in human cancers. In contrast to other tumor suppressors that are usually inactivated by frame-shift or nonsense mutations, the majority of cancer-associated TP53 mutations are non-synonymous missense substitutions, which indicate that cancer cells can benefit from the presence of mutated p53 protein with compromised functions (loss of function – LOF). Furthermore, some common TP53 missense mutations possess gain-of-function (GOF) properties that furnish mutant protein with new oncogenic qualities. A plethora of mutations detected in the TP53 gene, which gives rise to more than 2000 different protein variants, makes the issue very complex, but the progress in experimental and data mining methods provided significant advances in understanding functional consequences of different p53 mutations.

In this Research Topic, a few excellent reviews summarize the latest achievements in this field. Zhu et al. outline the spectrum of tumorigenic activities displayed by mutant p53 and discuss different therapeutic strategies to target cancer with TP53 mutations. The key targeted approaches – small molecule reactivators, gene editing, and immunotherapy aimed at mutant p53 tumors are also the topic of the review by Chasov et al. The work of Alvarado-Ortiz et al. is focused on the molecular mechanisms underlying the oncogenic activity of GOF p53 mutants. Based on the recent publications they analyze the role of p53 GOF mutations in metastasis, immune escape, metabolic reprogramming, cancer cell plasticity, and therapy resistance, and consider therapeutic perspectives. Metabolic functions of mutant p53 are particularly addressed by Etichetti et al. The authors recap current evidences for the role of mutant p53 in alteration of the mevalonate pathway and its contribution to enhanced prenylation of oncogenic proteins *via* positive regulation of Isoprenylcysteine Carboxyl Methyltransferase (ICMT). The interplay between mutant p53 and mevalonate pathway is further addressed in the research of Romeo et al. They explore how inhibition of STAT3 affects the mevalonate pathway and influences the expression of HSP90. The authors provide data arguing for the role of STAT3 in the stabilization of mutant p53 protein. On the other hand, it is known that in colorectal cancer the GOF hot-spot p53^R248Q/W^ mutants can positively regulate STAT3 activity *via* direct binding and protecting it from inhibitory dephosphorylation. Klemke et al. dissect the role of these and other mutant p53 variants in pancreatic cancer. Using a panel of PDAC cell lines they uncover a specific function of the p53^R248W^ mutant for enhancing the STAT3 axis which promotes migration of cancer cells. This study highlights p53 mutation-specific oncogenic features and underlines the functional heterogeneity of mutant variants. Hu et al. explore the role of TP53 mutations in PDAC using publically available gene expression data from patient samples. They uncover a link between mutant p53, small nuclear RNA (snoRNA)-mediated maturation of ribosomal RNA (rRNA), and PDAC progression: tumors with mutant p53 display increased snoRNA and rRNA levels, which correlates with poor prognosis. The relevance of TP53 mutational variability in cancer is in the focus of the review of Monti et al. The impact of p53 mutant variants for Chronic Lymphocytic Leukemia (CLL) is discussed here from the clinical perspective. Although TP53 mutations in CLL are rare and detected in less than 10% of cases at initial diagnosis, leukemia with compromised p53 displays faster progression and poor response to chemo- and immunotherapy. Unlike CLL, high-grade ovarian serous cancer (HGSOC) is characteristic of extremely high frequency (about 95%) and large variability of p53 mutations. Boyarskikh et al. analyze the spectrum of TP53 alterations in HGSOC patients with BRCA1/2 mutations. When looking for the association between BRCA1/2 and TP53 status the researchers find an unexpectedly increased proportion of TP53 truncations. Interestingly, almost 50% of detected p53 variants with missense mutations fall into a poorly characterized “unclassified” group with no clear LOF or GOF characteristics. p53 mutations affect not only classical tumor-suppressive mechanisms such as apoptosis or cell cycle arrest but can have an impact on other processes also relevant for tumor suppression. The role of wild-type p53 in the regulation of autophagy is well established, but it is less known how p53 mutations affect this process. Shi et al. provide a review of recent publications in this field and discuss how autophagy can be used for targeting mutant p53 in cancer. The latest progress in the understanding of the relationship between p53 activity and stemness is addressed in the review of Ghatak et al. The authors discuss how p53 controls pluripotency in normal cells and how oncogenic TP53 mutations drive cancer metastasis, cell plasticity, and resistance to therapy. Identification of mechanisms underlying chemoresistance associated with functional inactivation of p53 is an important task for the development of effective therapy. Deng et al. describe a p53-dependent mechanism that confers drug resistance in non-small lung cancer (NSCLC). Peroxisome proliferator-activated receptor coactivator (PGC1α) is essential for mitochondrial biogenesis and function, but its overexpression in NSCLC compromises the efficacy of platinum-based therapy and correlates with worse survival. Authors suggest that wild-type p53 is involved in the regulation of PGC1α protein stability, thus making cancer cells sensitive to chemotherapy. Tumor stroma can also contribute to drug resistance. Cancer cells induce changes in surrounding fibroblasts, endothelial and immune cells to adjust the microenvironment and make it favorable for tumor growth and dissemination. This is mediated by altered secretome and surface molecules produced by cancer cells. Besides cell-autonomous effects, p53 mutations can enhance the influence of cancer cells on the microenvironment. Capaci et al. provide a comprehensive review of recent publications dealing with the role of mutant p53 in shaping the tumor stroma.

Despite over 40 years of extensive research and myriad publications, our understanding of the role of p53 mutations in cancer development and therapy response is by far not complete. The papers collected in this Research Topic elucidate many important cellular mechanisms affected by p53 mutations and provide an outlook for the clinical consequences in different cancers. Together the reviews and experimental articles presented here have a significant impact on the field of p53 research.

## Author Contributions

The author confirms being the sole contributor of this work and has approved it for publication.

## Conflict of Interest

The author declares that the research was conducted in the absence of any commercial or financial relationships that could be construed as a potential conflict of interest.

## Publisher’s Note

All claims expressed in this article are solely those of the authors and do not necessarily represent those of their affiliated organizations, or those of the publisher, the editors and the reviewers. Any product that may be evaluated in this article, or claim that may be made by its manufacturer, is not guaranteed or endorsed by the publisher.

